# Fully Automated Deep Learning Enabled Miniature Mass Spectrometry System for Psychoactive Therapeutic Drug Monitoring

**DOI:** 10.1002/advs.202502721

**Published:** 2025-05-23

**Authors:** Yuanhao Zhou, Jiawen Ai, Zi Ye, Kexin Chen, JingXiong Lin, Zhenhua Zhang, Mi Luo, Benjie Zhou, Shijian Xiang, Jianhua Zhou, Xinming Huo

**Affiliations:** ^1^ School of Biomedical Engineering Shenzhen Campus of Sun Yat‐sen University Shenzhen 518107 China; ^2^ Key Laboratory of Sensing Technology and Biomedical Instruments of Guangdong Province School of Biomedical Engineering Sun Yat‐sen University Guangzhou 510275 China; ^3^ Division of Advanced Manufacturing Graduate School at Shenzhen Tsinghua University Shenzhen 518055 China; ^4^ State Key Laboratory of Precision Measurement Technology and Instruments Department of Precision Instrument Tsinghua University Beijing 100084 China; ^5^ School of Pharmaceutical Sciences (Shenzhen) Shenzhen Campus of Sun Yat‐sen University Shenzhen 518107 China; ^6^ Shenzhen Key Laboratory of Chinese Medicine Active Substance Screening and Translational Research Department of Pharmacy, The Seventh Affiliated Hospital Sun Yat‐sen University Shenzhen 518107 China; ^7^ CHIN Instrument (Hefei) Co., Ltd. Hefei 231200 China

**Keywords:** clinical mass spectrometry, therapeutic drug monitoring, automated analysis, miniature mass spectrometer, intelligent diagnosis

## Abstract

Advancing precision medicine requires efficient small molecule biomarker detection in biofluids, yet existing methods encounter challenges in complexity, portability, and throughput. This study presents an integrated miniature blood processing and mass spectrometry (MS) analysis system, which incorporates automated magnetic solid‐phase extraction, self‐aspiration sampling miniature mass spectrometer, and deep learning algorithms for automated quantitative analysis. It achieves full automation from sample preparation to detection, demonstrating the capability to analyze serum psychoactive drugs with a 15‐second/MS acquisition and 8‐sample parallel processing within 30 minutes (including pretreatment). This has significantly increased detection throughput and facilitated the establishment of the standard curve. The novel dual‐target ion parallel tandem MS analysis technique, combined with a U‐net peak area recognition algorithm, achieved over 98% identification accuracy with less than 0.2% area prediction deviation. Quantitative analysis showed high correlation coefficients >0.99 in medically relevant ranges, supported by relative standard deviation < 10% and average back‐calculated accuracy deviation < 3.5%. Clinical validation revealed strong concordance with LC‐MS/MS. The system's integration of automated sample processing, miniature MS hardware, and AI‐driven data analysis establishes a paradigm for high‐throughput clinical detection. The advantages of accuracy, automation, intelligence, miniaturization, and high throughput suggest significant potential for this system in clinical detection and personalized medicine.

## Graphical abstract

This study presents a fully automated miniature mass spectrometry system for high‐throughput therapeutic drug monitoring. Integrating magnetic solid‐phase extraction, self‐aspiration sampling miniature mass spectrometer, and U‐net deep learning quantitative algorithm, it achieves rapid (8 samples/30 min), precise (R^2^ > 0.99, RSD < 10%), and portable analysis. Validated for psychoactive drugs, it shows strong clinical correlation with LC‐MS/MS, enabling intelligent, miniaturized, and automated point‐of‐care diagnostics.

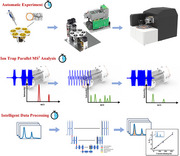

## 1 Introduction

Biological fluids analysis is a key approach in biomedical applications, pharmacological research, and biomarker identification.^[^ ^1^ ^]^ Compared to tissue samples, biological fluids such as blood and urine are more readily obtainable and can reflect the body's circulatory profile and overall condition. Blood sample collection is a routine part of almost all standard medical examinations, and current clinical needs for various biomarkers detection^[^ ^2^, ^3^ ^]^ and therapeutic drug monitoring (TDM)^[^ ^4^, ^5^ ^]^ are primarily addressed through blood tests. From a technical perspective, commonly used blood analysis methods in clinical practice include high‐performance liquid chromatography (HPLC),^[^ ^6^ ^]^ liquid chromatography‐tandem mass spectrometry (LC‐MS/MS),^[^ ^7^ ^]^ immunoassays,^[^ ^8^ ^]^ electrochemical methods,^[^ ^9^ ^]^ and biosensor techniques.^[^ ^10^ ^]^ Among these technologies, LC‐MS/MS has emerged as the gold standard for measuring various metabolic biomarkers and small‐molecule drug concentrations in blood, owing to its high specificity and sensitivity.^[^ ^11^, ^12^ ^]^ Despite this, due to the interference of the strong matrix effect and stringent requirements for accuracy and stability, the widespread application of clinical mass spectrometry (MS) still encounters substantial obstacles, specifically in terms of automation, intelligence, miniaturization, and detection throughput.

First of all, because of the strong matrix interference effect of blood, sample pretreatment turns into an essential step. These methods often involve discrete and multi‐step offline workflows, serving as a major source of error in analytical processes. Therefore, the automation of pretreatment steps is of crucial importance for enhancing the repeatability, reliability, and efficiency of the analytical process.^[^ ^13^, ^14^, ^15^ ^]^ Automated sample preparation systems can perform various sample handling tasks, such as pipetting, mixing, diluting, derivatizing, and extracting.^[^ ^16^, ^17^ ^]^ They offer higher throughput, repeatability, and accuracy, and can be programmed to execute complex workflows without manual intervention, thereby improving the precision and accuracy of the final analytical results.^[^ ^18^ ^]^ Today, various types of automated systems with different architectures and complexities provide effective support for applications in analytical chemistry. Although these automated preprocessing devices have gradually been applied to LC‐MS/MS methods,^[^ ^19^, ^20^ ^]^ integrated devices that combine preprocessing and analysis are still rarely reported.

Furthermore, as a highly precise analytical instrument, the mass spectrometer typically requires trained professionals to operate it and process the data. With the increase in real‐time data acquisition rates of modern instruments, the amount of data has increased exponentially. This demands a significant amount of time and effort from experts to process, interpret, and convert the data, which will also lead to unnecessary errors. In recent years, with the advancement of artificial intelligence technologies, various machine learning algorithms have been deeply integrated into analytical tools. For example, machine learning methods have been applied to LC‐MS,^[^ ^21^ ^]^ GC‐MS,^[^ ^22^ ^]^ NMR,^[^ ^23^ ^]^ and X‐ray diffraction^[^ ^24^ ^]^ for real‐time data analysis, enhancing their capability for auxiliary detection. Moreover, intelligent data processing not only reduces reliance on specialists but also automatically corrects potential errors during data handling, improving the overall reliability of the analysis.^[^ ^25^, ^26^ ^]^ However, current artificial intelligence algorithms associated with MS data predominantly concentrate on qualitative identification.^[^ ^27^, ^28^, ^29^ ^]^ Quantitative data processing remains inefficient, especially for detecting small‐molecule biomarkers in blood. Constructing calibration curves typically requires processing 6–8 concentration points and multiple test samples, involving signal extraction, averaging, and ratio calculation. This manual workflow is time‐consuming and labor‐intensive, particularly for miniature mass spectrometers, which often lack automated and user‐friendly data processing tools.

Moreover, with the continuous advancement of precision medicine and personalized treatment, the demand for bedside testing and high‐throughput analysis is increasing. Traditional laboratory mass spectrometers, owing to their large size, high costs, and the need for specialized personnel for maintenance, limit their widespread application in clinical testing. In the past two decades, the rapid development of miniaturization technologies for mass spectrometers has made point‐of‐care testing (POCT) in clinical MS possible.^[^ ^30^, ^31^, ^32^ ^]^ The progress in ambient ionization techniques enables the direct, rapid, and real‐time MS analysis, exhibiting substantial potential in clinical sample examination.^[^ ^33^, ^34^, ^35^ ^]^ Moreover, the compact design and simplification of miniature mass spectrometers result in significantly lower manufacturing costs compared to those of traditional large‐scale mass spectrometers. In addition, eliminating the need for an LC system further reduces both equipment and maintenance costs, making it a more economically advantageous option. Despite these advancements, the existing technologies are still insufficient in scenarios that require high quantitative accuracy and stability, such as the detection of the concentrations of small molecular markers in blood. Therefore, the mainstream approach for clinical blood testing currently still relies on LC‐MS/MS.^[^ ^12^ ^]^ However, chromatography, as a single‐channel separation technique, has limitations in terms of timeliness and throughput. Specifically, establishing quantitative standard curves with current LC‐MS/MS methods often requires 1–2 h of processing time, which restricts the application of MS technology in high‐throughput testing. Although the advent of ultra‐performance liquid chromatography (UPLC) can speed up the separation process, the high cost of instruments and consumables still limits its widespread use.

To address the limitations and challenges faced by clinical mass spectrometry, we propose an integrated miniature blood processing and mass spectrometry analysis system (imBPMS). This system combines automated magnetic solid‐phase extraction preprocessing, self‐aspiration sampling miniature mass spectrometer, and deep learning algorithms for automatic quantitative analysis. By introducing magnetic nanoparticles with specific affinity for the target analytes, we developed an automated magnetic solid‐phase extraction (MSPE) workflow. It effectively removes matrix interferences and enriches analytes, minimizing serum matrix effect. Compared to the conventional SPE method, magnetic particles allow rapid separation and elution via magnetic force, eliminating the need for column packing and facilitating integration into automated systems. By directly coupling the sample introduction interface of the miniature ion trap mass spectrometer with the preprocessing module, the system enables high‐throughput, automated preprocessing and analysis of blood samples without the need for chromatographic separation. Additionally, the study implements a dual‐target ion parallel tandem mass spectrometry (MS^2^) analysis method and a peak area recognition algorithm based on U‐net deep learning theory to achieve automatic peak area recognition and quantitative analysis. On this basis, experiments to detect psychoactive therapeutic drugs in serum were conducted to characterize the system's performance. And due to their widespread clinical use and narrow therapeutic windows in the treatment of psychiatric disorders, venlafaxine, desvenlafaxine, risperidone, and 9‐hydroxyrisperidone were selected as representative targets. This research integrates the entire workflow from sample preprocessing to MS analysis and data interpretation. System characterization and clinical experiments demonstrate the advantages of this system in automation, intelligence, miniature, and high‐throughput capabilities, ensuring accurate monitoring of target analytes while achieving simplicity, portability, and low cost in the detection system, thereby maximizing the medical assistance potential of a miniature mass spectrometer for patients.

## 2 Experimental Section

### 2.1 Chemical and Regent

Methanol, acetonitrile, and formic acid (chromatographic grade) were procured from Merck KGaA (Germany). Ultrapure water was obtained from China Resources C'estbon Beverage (China) Co., Ltd. Silica capillaries used for sub‐atmospheric pressure self‐aspiration capillary electrospray ionization were purchased from Polymicro Technologies (Phoenix, AZ).

Calf serum was sourced from America AMRC (Jiangsu, China). Actual patient samples were provided by the Seventh Affiliated Hospital, Sun Yat‐sen University. The relevant clinical trials have been reviewed by the medical ethics committee of the Seventh Affiliated Hospital of Sun Yat‐sen University (Shenzhen) (No. KY‐2024‐206). C18 magnetic nanoparticles were supplied by ADM Technology (Nanjing, China). Venlafaxine, Desvenlafaxine, Risperidone, 9‐hydroxy Risperidone, and their internal standards (Venlafaxine‐d6, Desvenlafaxine‐d6, Risperidone‐d4, 9‐hydroxy Risperidone‐d4) were all acquired from Anpel (Shanghai, China). Stock solutions of the four standards (1 mg mL^−1^) and their corresponding internal standards (100 µg mL^−1^) were pre‐prepared in methanol and stored at −20 °C until use.

### 2.2 Instrument Introduction

**Figure 1:**
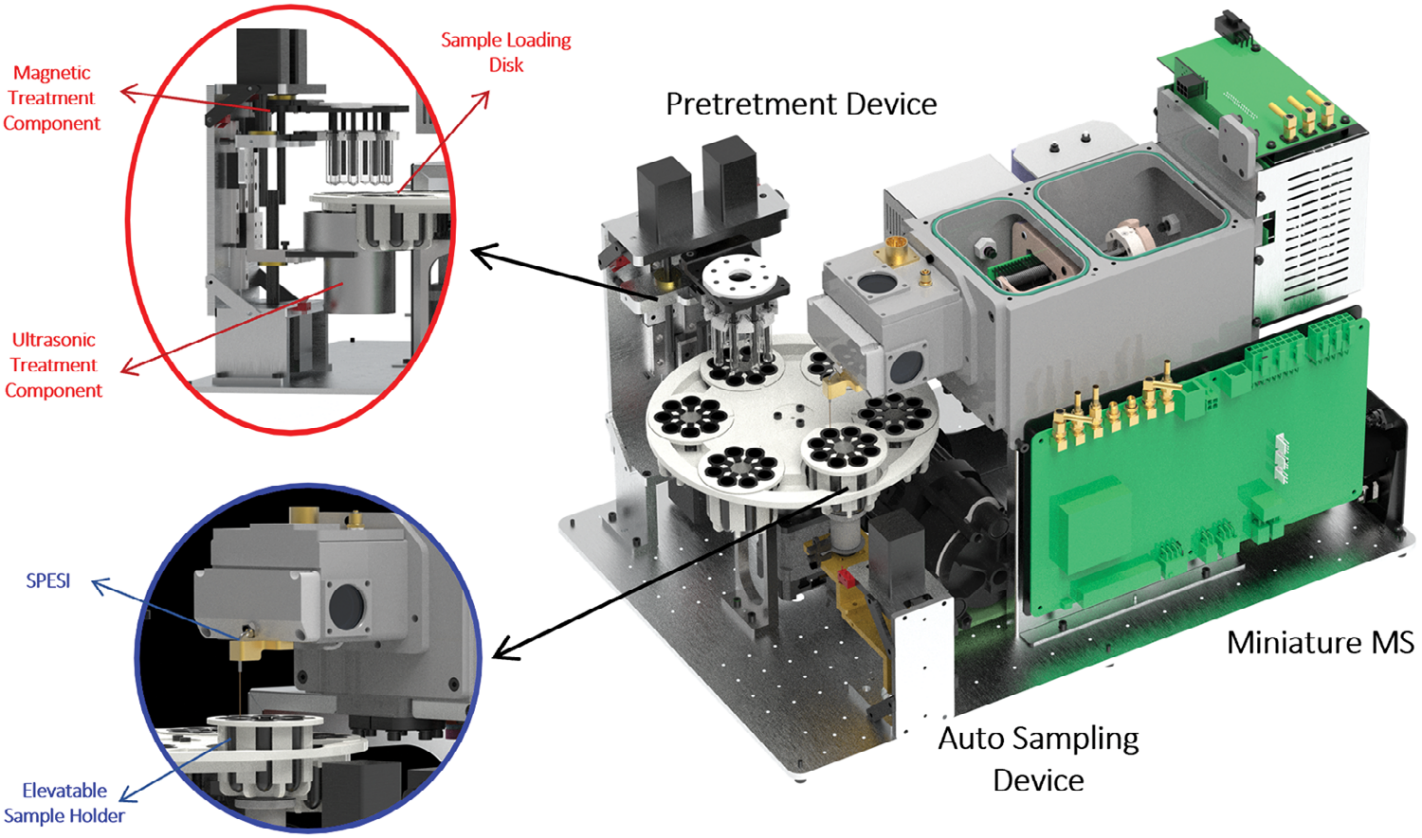
Schematic diagram of the imBPMS.

The integrated miniature blood processing and mass spectrometry analysis system (imBPMS) developed in this study is structured as shown in **Figure** **1**. It can be divided into three main components: the pretreatment device, the auto sampling device, and the miniature MS device.

The pretreatment device primarily functions to achieve the selective extraction of target drugs from serum samples. The device consists of a sample loading disk and two pretreatment components. The sample loading disk is embedded with six groups of small disc‐type centrifuge tube holders, enabling the automation of six sample processing steps. Each centrifuge tube holder can accommodate eight centrifuge tubes, allowing for simultaneous parallel processing of eight sample sets. The pretreatment components include a magnetic treatment component made up of a magnetic rod and a magnetic rod sleeve, as well as an ultrasonic treatment component. The magnetic rod, its sleeve, and the ultrasonic water bath are all capable of vertical movement. The magnetic rod sleeve can mix and disperse the samples through vertical vibrations, while the ultrasonic treatment component transmits ultrasound to the samples via the water bath, facilitating auxiliary processing. Upon the addition of samples to the sample loading disk, the centrifuge tube holders rotate sequentially to the position of the treatment components for dispersion and transfer. This process ultimately results in the processed sample solution.

After the sample processing is completed, it is introduced into the mass spectrometer for direct detection through the automatic sampling device. The sample introduction device consists of an elevatable centrifuge tube holder and a sub‐atmospheric pressure self‐aspiration capillary electrospray ionization source (SPESI).^[^ ^27^ ^]^ The centrifuge tube holder was driven by a motor to rise to a designated position, allowing the capillary to be inserted into the centrifuge tube solution for online sampling. To address the limited sampling efficiency of self‐aspirating capillary electrospray sources,^[^ ^28^ ^]^ the spray end of the capillary for SPESI is inserted into a sub‐atmospheric pressure chamber, while the inlet end remains at atmospheric pressure. The capillary is secured to the chamber using a custom‐designed hollow sealing connector. The driving force generated by the pressure differential facilitates the self‐aspirating sampling function. The entire pretreatment and sample introduction process is fully automated, requiring no manual intervention.

For the MS device, a miniature mass spectrometer previously developed by our research group was utilized, which features a continuous sub‐atmospheric pressure interface and an integrated ionization source. The instrument comprises a sub‐atmospheric pressure spray chamber, an ion funnel transmission chamber, and an ion trap analysis chamber. And its main parameters have been described in earlier publications.^[^ ^27^, ^36^ ^]^ To facilitate the installation of the automated sample introduction device, the layout of the instrument was modified as the latest structure shown in Figure 1. To prevent micro‐impurities in the samples from clogging the spray capillary, a capillary made of fused quartz was employed with a relatively large inner diameter (outer diameter: 360 micrometers, inner diameter: 150 micrometers, tip inner diameter: 30 micrometers). The temperature of the ion introduction tube is maintained at ≈200 °C to keep the pressure within the ion trap chamber in the range of 0.1–0.15 Pa.

### 2.3 Pretreatment Method

During the sample pretreatment process, magnetic solid‐phase extraction (MSPE) principles were employed,^[^ ^37^, ^38^ ^]^ using magnetic nanoparticles modified with high‐density C18 chains (shown in Figure S1, Supporting Information) for the selective extraction of psychoactive compounds from serum. This process is fully automated with the developed device. By placing the pre‐prepared solutions in the centrifuge tubes, the entire process can be automatically completed. The workflow consists of six steps: 1) Activation: disperse 6 mg of Fe_3_O_4_@C18 magnetic nanoparticles in 1 mL of methanol‐water for 1 min; 2) Adsorption: use an external magnetic rod to transfer the nanoparticles to 400 µL of diluted serum (180 µL water, 180 µL serum, and 40 µL internal standard) and disperse for 5 min; 3) Washing: transfer the nanoparticles to 1 mL of ultrapure water and wash for 1 min; 4,5) Repeat the washing process twice; 6) Elution: transfer the nanoparticles to 400 µL of 80% methanol‐water and disperse for 4 min to elute the target compounds, then remove the magnetic nanoparticles to obtain the remaining eluent for analysis. The entire pretreatment workflow can be completed within 25 min. In the above process, the mixing of nanoparticles and solution is driven by the vibration of the magnetic rod sleeve, and ultrasonic baths assist in all steps except washing.

It was important to note that two methods were employed for adding the internal standard in these experiments. In routine sample testing, the internal standard was added to the serum solution during step two. In contrast, for experiments investigating the impact of pretreatment conditions on MSPE adsorption performance, the internal standard was added to the elution solution in step six. This approach allowed for effective comparison of process efficiency (PE) under different pretreatment schemes.

### 2.4 Intelligent Quantitative Analysis Method

Monitoring drug concentrations in blood imposes high demands on the quantitative capabilities of detection methods. Traditional LC‐MS/MS techniques utilize the peak height of specific ions in the MS signal as the detection signal for target substances, while the integrated peak area is used to establish quantitative standard curves, resulting in highly accurate and stable quantitative performance.^[^ ^39^ ^]^ To enhance detection throughput, reduce instrument size, and lower costs, this study eliminates the chromatography separation step and directly employs the detection results from a miniature ion trap mass spectrometer for quantitative analysis. However, because the ion trap operates on a sampling‐then‐detection principle, its quantitative stability has always been inferior to that of a quadrupole mass spectrometer.^[^ ^40^ ^]^ Consequently, it poses significant challenges for the quantitative methods of this system. To address this issue, a novel quantitative scheme was proposed that combines a dual‐target ion parallel MS^2^ analysis method with a U‐net deep learning peak area recognition algorithm.

**Figure 2:**
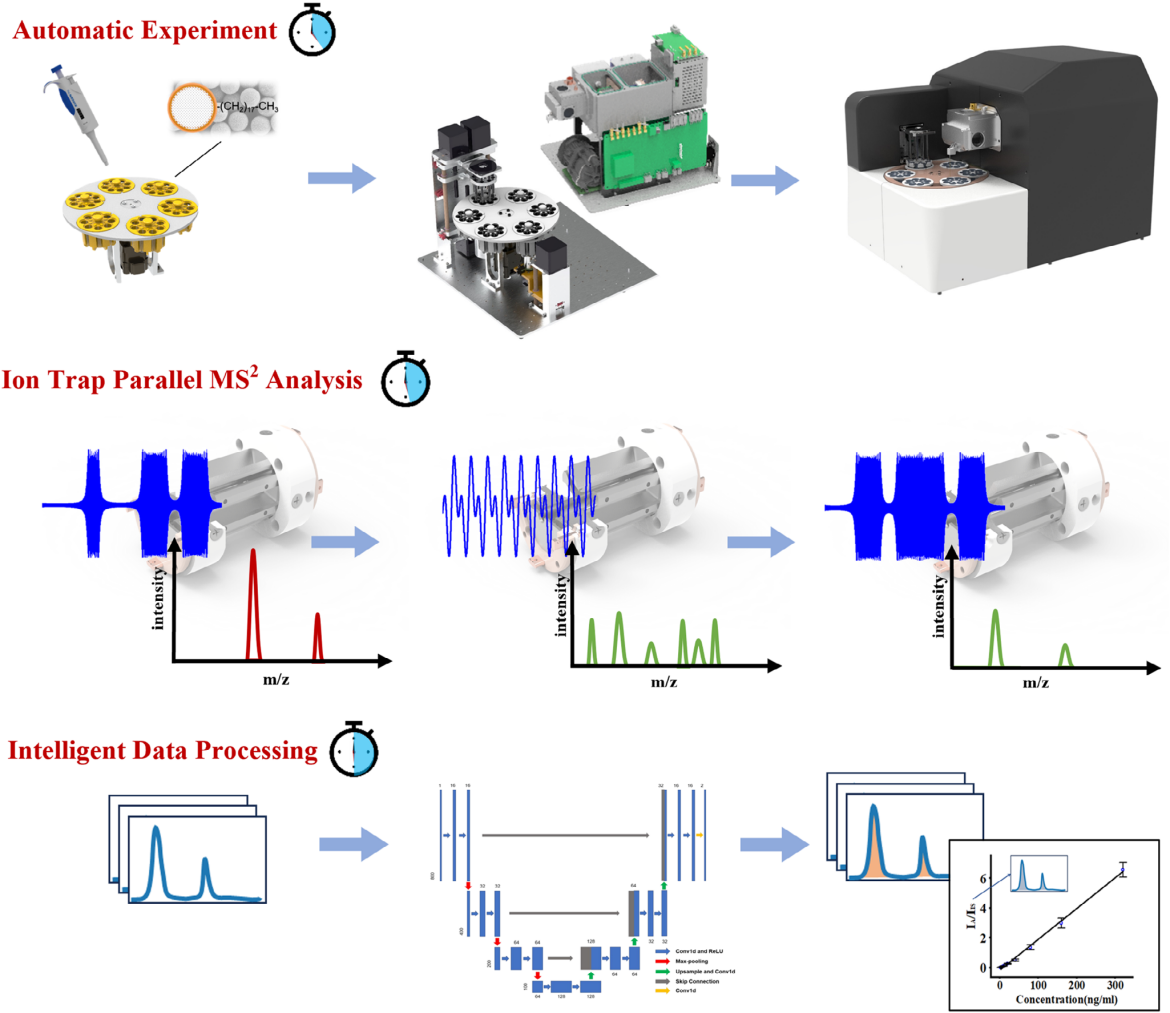
Schematic diagram of the detection process.

As shown in **Figure** **2**, a stable isotope internal standard with a specified concentration is introduced during the pretreatment process. The internal standard shares similar properties and ionization efficiency with the target substance, thereby effectively eliminating system interference to the greatest extent. To mitigate injection errors among multiple analyses in the ion trap mass spectrometer, the previously established high‐resolution ion isolation technique was improved.^[^ ^41^ ^]^ After a single ion injection, ions of the target substance and its isotope internal standard are simultaneously isolated within two distinct mass windows. Subsequently, a dual‐frequency auxiliary excitation signal is employed to trigger synchronous collision‐induced dissociation (sCID) of the two precursor ions. Following the sCID, a distinctive fragment ion is chosen for both the target analyte and its internal standard, with the peak area ratio of these two fragment ions serving as the basis for subsequent quantitative analysis. Finally, a quantitative curve is constructed by performing linear fitting between the concentration of the spiked samples and the peak area ratios.

Here, in order to overcome the instability of the miniature mass spectrometer and enable automatic identification of quantitative results, a U‐net deep learning algorithm was developed to recognize and calculate the peak areas of the two target fragment ions. As shown in Figure 2, the U‐net architecture consists of three layers of down‐sampling and three layers of up‐sampling, utilizing cross‐layer connections to extract and integrate features at different levels, thereby enabling precise segmentation of peak shapes. After acquiring the mass spectrum, signal intensity segments were selected for the target m/z along with two mass units on either side. These segments are resampled at 0.01 m/z intervals to create input vectors $\vec{x}$ of length 400. During model training, expert annotations are utilized to identify the left and right endpoints of the target peak. The signal within the target peak range is labeled as 1, while all other signals are labeled as 0, generating a corresponding label vector $\vec{y}$ of length 400. The peak area can then be calculated as follows:

(1) $$S=(\vec{x}\cdot \vec{y})\times ▵m$$

where $\vec{x}$ and $\vec{y}$ represent the intensity vector of the target fragment ion and the corresponding 0–1 label vector, respectively. While ▵*m* denotes the m/z interval between adjacent points in the sequence, which is set as 0.01.

To verify the accuracy of the U‐net algorithm in identifying peak areas and to characterize the quantitative effects of the method, four psychoactive therapeutic drugs were selected, along with their isotopic internal standards as test samples. The selected characteristic ion parameters are listed in Table S1 (Supporting Information). A total of 727 samples with varying concentrations were collected, of which 490 were used for training and 237 for testing. For each sample, two characteristic peaks were extracted for the target substance and its isotopic internal standard, resulting in 980 pairs of spectral peaks and 392,000 data points used for training the U‐net network. The trained U‐net model was then used to predict peak areas for the 237 samples. Here, the algorithm's peak shape recognition capability was compared with manually annotated peaks. The manual annotations were performed by experienced analysts, who accurately marked the start and end points of each peak and then calculated the peak area through numerical integration over the manually defined region. Subsequently, the ratio between the algorithm‐predicted peak area and the manually annotated peak area was calculated and used as the primary metric to evaluate the accuracy of the algorithm.

### 2.5 Statistical Analysis

All statistical analyses were conducted using Python, primarily utilizing the SciPy and statsmodels libraries. Experimental data are presented as mean ± standard deviation (SD). For optimization experiments in the pretreatment process, the sample size for each condition was *n* = 4, corresponding to measurements from four different analytes. No data transformation or normalization was applied prior to analysis. For comparisons involving multiple experimental conditions (such as the amount of magnetic nanoparticles, types of washing reagents, number of washing steps, and types and volumes of elution solvents), one‐way analysis of variance (One‐way ANOVA) was used to assess statistical significance. For paired experimental designs (e.g., comparison between 200 and 400 µL elution volumes), a paired *t*‐test was employed. All tests were two‐tailed, and a significance level of α = 0.05 was used. Differences were considered statistically significant when *P* < 0.05.

## 3 Results and Discussion

### 3.1 Optimization of Pretreatment Methods

In this study, we conducted an investigation into the impact of various parameters on the efficiency during the pretreatment process. The process efficiency (PE) is calculated based on the response ratio of the target analytes to the internal standard after the serum samples undergo the entire analysis process, compared to the response ratio obtained when the target analytes are dissolved in pure solvent. And these parameters encompassed the amount of magnetic nanoparticles, the type of washing reagents, the type and amount of eluent solvent, as well as the duration of the adsorption process. All conditions specified in Section 2.3 were applied during the experiments, except for the parameters to be optimized. The optimization process utilized spiked calf serum to simulate real patient serum, with both internal standard and spiked concentrations set at 100 ng mL^−1^, which provided a strong and reliable response in MS analysis.

#### 3.1.1 Amount of Magnetic Nanoparticles

**Figure 3:**
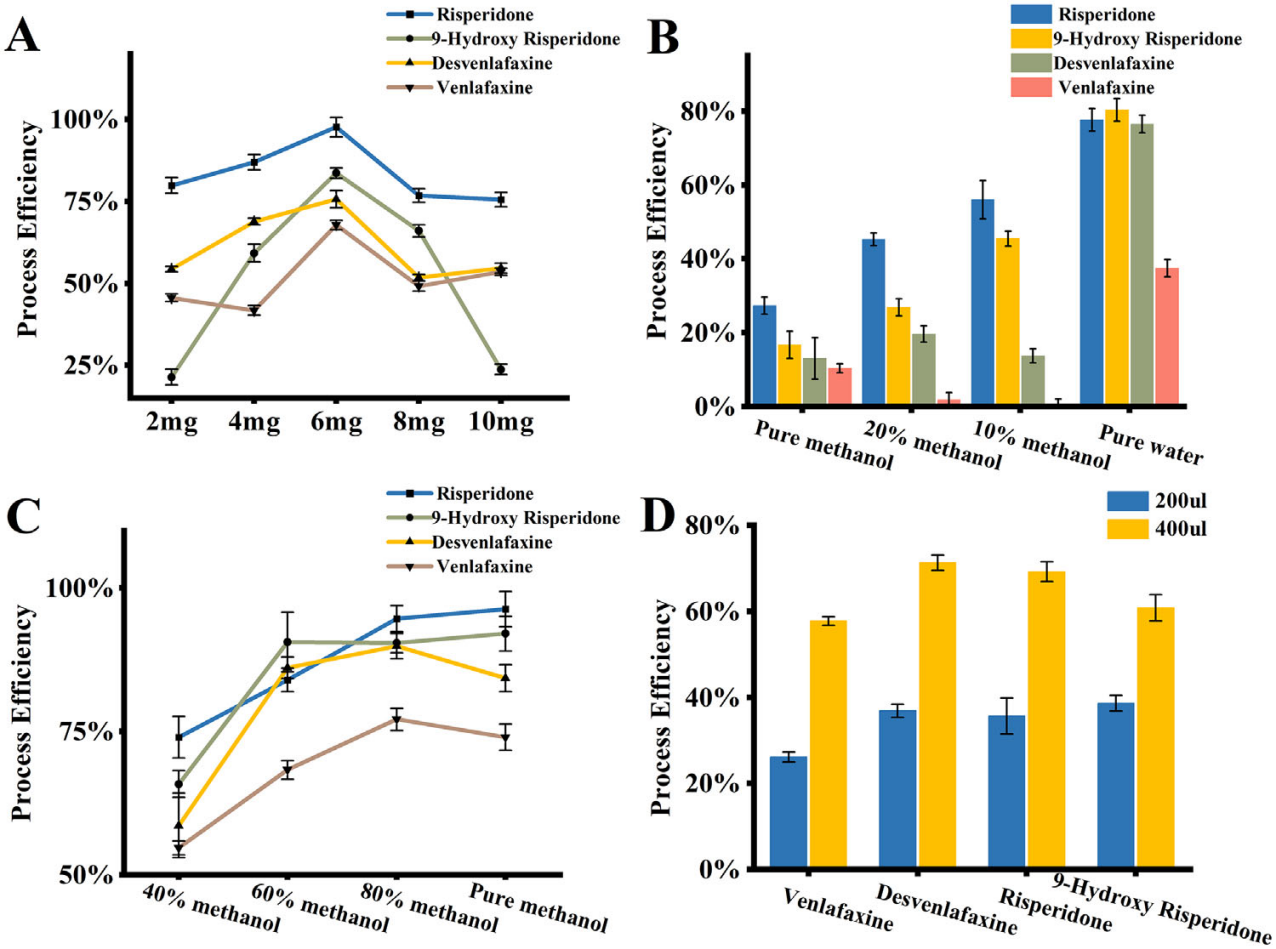
Optimization results of experimental conditions for the process efficiency. A) Effect of the amount of magnetic nanoparticles; B) Effect of the type of washing reagents; C) Effect of the type of elution solvent; D) Effect of the volume of elution solvent.

Target analyte adsorption is the most critical step influencing extraction efficiency, and the amount of adsorbent is a key parameter directly affecting both extraction efficiency and cost. We conducted experiments using 2, 4, 6, 8, and 10 mg of magnetic nanoparticles to evaluate the process efficiency for the four drugs. The results depicted in **Figure** **3**A demonstrate that the process efficiency exhibits a trend of increasing and then decreasing with the increase in nanomaterials. One possible reason is that the adsorption effect of magnetic nanoparticles increases with the increase in the amount used. However, after reaching the saturation effect, the dispersibility of the nanoparticles in the solution decreases, leading to a decline in their adsorption effect. Besides, the repeated measures one‐way ANOVA revealed a statistically significant influence of the adsorbent amount on PE performance (*P*< 0.022), indicating that variations in the amount of adsorbent markedly affected extraction efficiency. Among the tested conditions, the 6 mg group yielded the highest overall PE across samples, indicating it as the optimal amount for the experiments.

#### 3.1.2 Types of Washing Reagents and the Number of Washes

The purpose of the washing step is to remove as many impurities adsorbed on the surface of the magnetic nanoparticles as possible while retaining the target analytes. Different types of washing reagents exhibit varying effectiveness in mitigating matrix interference. We conducted comparative experiments using pure methanol, 20% methanol in water, 10% methanol in water, and pure water as washing reagents. The results, shown in Figure 3B, indicate that the addition of methanol leads to a decrease in process efficiency, likely due to its strong elution capability, which causes loss of the target analytes. Besides, the repeated measures one‐way ANOVA revealed a statistically significant influence of the washing reagent type on PE (*P* < 0.001). Among the tested conditions, pure water yielded the highest PE, suggesting its superior performance in impurity removal while preserving the target analyte, and therefore was selected for subsequent experiments. Meanwhile, we also observed that after a single wash with pure water, a small amount of foam appeared in the solution, indicating that a single water wash was insufficient to completely remove proteins and peptides from the nanoparticle surface. Further experiments revealed that after three washes with water, the foaming phenomenon ceased. Consequently, we chose to perform three consecutive water washes during the washing step.

#### 3.1.3 Types and Amount of Elution Solvents

As mentioned earlier, adjusting the ratio of methanol to water can modify the polarity of the reagents, thereby affecting the elution efficiency. In the experiments, we prepared elution solvents with varying methanol‐water ratios of 40%, 60%, 80%, and pure methanol. The results, shown in Figure 3C, indicate that an increased proportion of methanol in the elution liquid facilitates the elution of target analytes. The PE under four elution conditions were also compared using repeated measures ANOVA. The analysis revealed a statistically significant effect of elution solvent on PE (*P* < 0.001). However, considering that methanol is a strong polar solvent, its strong elution capability may also remove other impurities that are strongly adsorbed to the magnetic nanoparticles, leading to significant matrix effects. For instance, pure methanol elution resulted in baseline drift, as illustrated in Figure S2 (Supporting Information). Furthermore, based on the characteristics of self‐aspirating electrospray, excessively high water content in the eluent can negatively affect desolvation.^[^ ^42^ ^]^ Therefore, we ultimately selected 80% methanol in water as the elution solvent.

The volume of the elution solvent also impacts the concentration factor in the pretreatment process. We compared the process efficiency using 200 and 400 µL of elution solvent. As shown in Figure 3D, the use of 400 µL of elution solvent yields better process efficiency. Although the 400 µL elution solvent results in dilution compared to the original serum sample, leading to lower signal responses, the reduced matrix effects actually enhance process efficiency. A paired *t*‐test was performed to compare the PE under different elution volumes. The results showed a statistically significant increase in PE when 400 µL of elution solvent was used compared to 200 µL (*P* < 0.002). Additionally, selecting 400 µL of solution is more favorable for the subsequent online sampling in the automatic sampling device.

#### 3.1.4 Other Factors

Additionally, several other factors can influence the extraction efficiency of the magnetic nanoparticles. For instance, since most serum composition is water, the hydrophobic magnetic nanoparticles need to be activated with a polar organic reagent before entering the serum. We used 50% methanol in water as the activation reagent. To minimize the loss of target analytes during the pretreatment, we selected an excessive adsorption time (5 min) and an extended elution time (4 min) to ensure sufficient adsorption and elution.

### 3.2 Quantitative Method Characterization

Mass spectrometry is generally considered a high‐resolution analytical method compared to chromatography and ion mobility spectrometry, often using peak height to reflect the concentration of the detected substance for quantitative analysis. However, for miniature ion trap mass spectrometers, the simplification of the instrument and the higher operating pressure significantly reduce resolution, making it difficult to meet the demands of high‐precision quantitative analysis. For example, an ideal mass spectrum peak has a normal distribution shape (see Figure S3a, Supporting Information), where peak height and peak area can give similar quantitative results. However, unstable factors such as resonance excitation at higher pressures can introduce tailing effects (see Figure S3b, Supporting Information), making it difficult to capture this ion information using peak height detection. In addition, when low abundance signals are affected by baseline drift or noise interference, the mass spectrum peak may exhibit peak splitting (see Figure S3c, Supporting Information), making accurate peak height identification difficult. Thus, for miniature mass spectrometers with irregular or complex peak shapes, peak area quantification promises better identification results. In addition, relying on manual information collection and data processing is not only cumbersome, but also introduces a great deal of subjective uncertainty. To address these challenges, a dual‐target ion parallel MS^2^ analysis method was developed to overcome the instability of multiple injections for the ion trap. On this basis, a U‐net deep learning algorithm model was designed to automatically identify and learn the features of mass spectra peaks and calculate peak areas.

**Figure 4:**
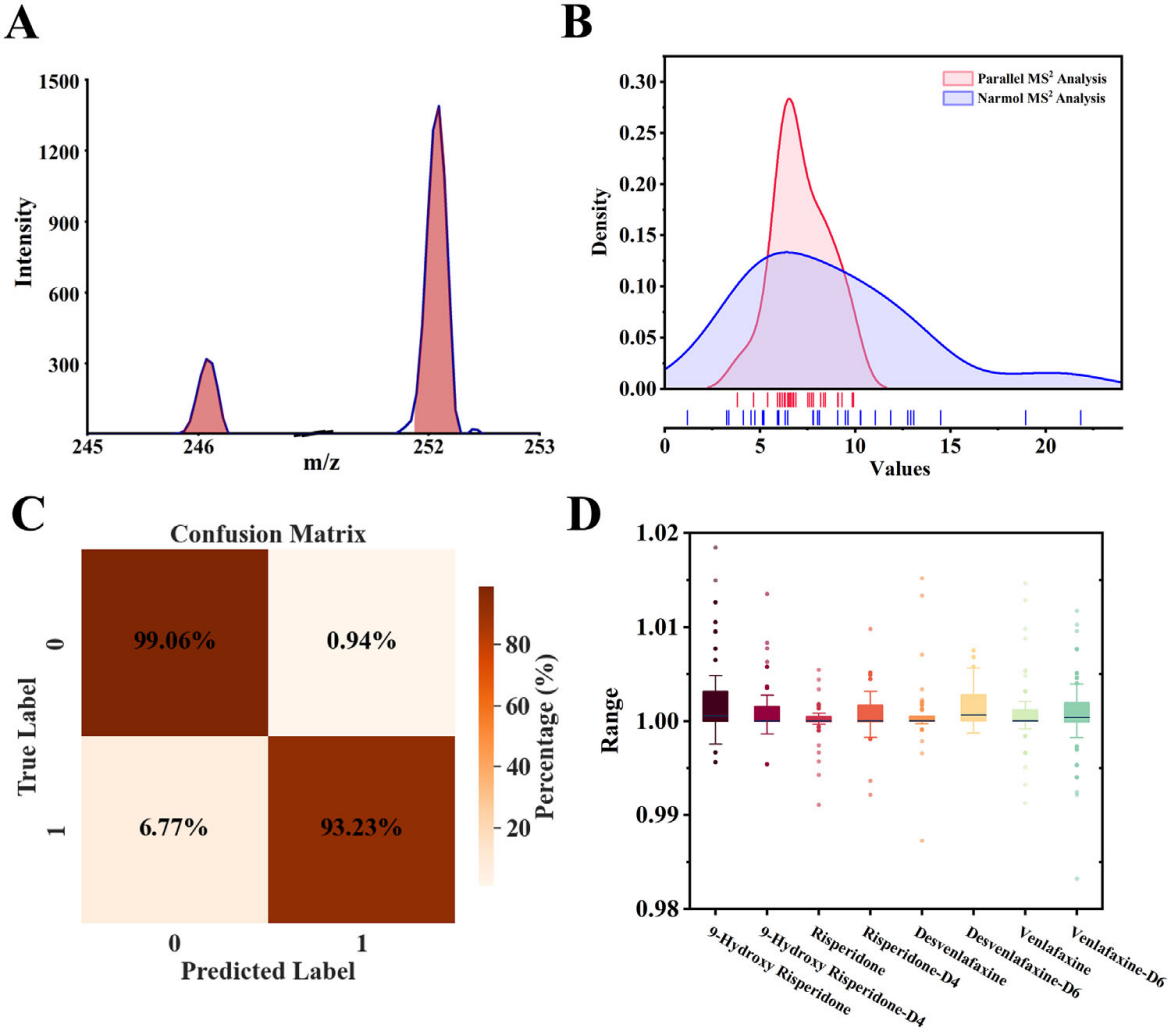
Characterization of the peak area recognition algorithm. A) The peak recognition result of the characteristic fragment ion pairs for Venlafaxine and its isotope internal standard. B) The effect of different isolation methods on the intensity ratio stability of two target ions. C) Confusion matrix of the recognition data derived from the test spectra. D) The comparison results of the deviation between the peak area predicted by the algorithm and the area as manually delineated.

**Figure** **4**A presents the peak recognition result of the characteristic fragment ion pairs for Venlafaxine and its isotope internal standard. To test the effect of the dual‐target ion parallel MS^2^ analysis method on the stability of the miniature mass spectrometer, we used parallel MS^2^ analysis and normal MS^2^ analysis to perform 30 consecutive tests on 9‐hydroxyrisperidone and its internal standard. The ratio of peak intensities obtained is shown in Figure 4B. The parallel MS^2^ analysis method showed a significantly more concentrated distribution of intensity ratios, demonstrating its advantage in improving quantitative stability. Then we selected a total of 237 samples from four psychoactive drugs and their internal standards, resulting in 189,600 data points for testing the accuracy of peak area recognition. The final identification results are displayed in the 2D confusion matrix shown in Figure 4C. The counts for true positives (TP), false positives (FP), true negatives (TN), and false negatives (FN) were 23,064, 163,104, 1,700, and 1,732, respectively. The calculation method is delineated in Table S2 (Supporting Information). The sensitivity of the U‐net algorithm in peak shape recognition was 99.06%, with a specificity of 93.22%, resulting in an overall accuracy of 98.3228%. This algorithm exhibited an excellent perfomance for peak shape recognition, thereby contributing significantly to subsequent quantitative analysis.

Furthermore, the predicted peak area values from the algorithm were compared with the manually marked peak areas by experts, as shown in Figure 4D. For the eight target peaks, the mean discrepancy between the predicted peak area values from the developed algorithm and the manually standardized peak area values was determined to be 0.196%. This negligible discrepancy suggests the high precision and reliability of the algorithm in predicting peak areas. Additionally, the algorithm enables a fully automated peak area recognition process, thereby eliminating the need for manual intervention. When integrated with the eight‐channel automatic pretreatment and automatic sampling device, a standard curve based on peak areas can be established in a single step for quantitative determination.

### 3.3 System Reliability Evaluation

The reliability of the system was assessed by evaluating the established method in terms of process efficiency, quantitative accuracy, and detection precision.

#### 3.3.1 Process Efficiency

**Figure 5:**
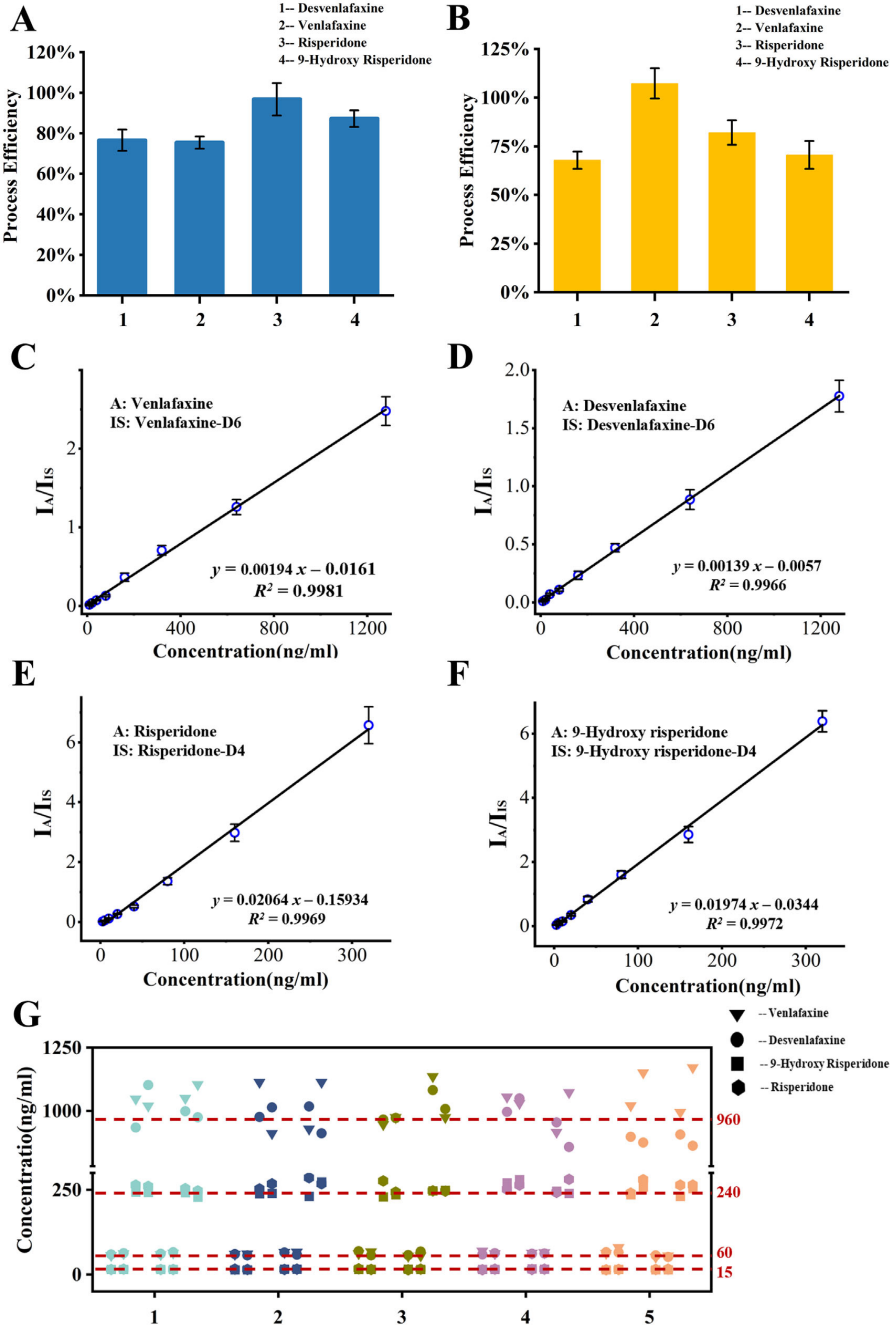
System reliability evaluation characterization. A) Process efficiency of the liquid–liquid extraction method; B) Process efficiency of the magnetic solid phase extraction method; C–F) Quantitative linear curves for venlafaxine, desvenlafaxine, risperidone, and 9‐hydroxyrisperidone, respectively; G) Precision test results for four drugs at high and low concentration levels.

The process efficiency serves as a pivotal metric for evaluating the efficacy of the pretreatment and analysis procedures, which exerts a direct influence on the reliability of the detected outcomes. Following the optimization of optimal pretreatment conditions, an assessment was conducted to determine the overall process efficiency of four psychoactive therapeutic drugs within a blank calf serum sample. A comparison was made between the MSPE method and traditional liquid–liquid extraction. The experimental results, as illustrated in **Figure** **5**A,B, demonstrate that the process efficiency for the four drugs using the MSPE method ranged from 60% to 120%, achieving levels comparable to those of traditional liquid–liquid extraction. A notable advantage of the MSPE method is its substantial reduction in the consumption of organic reagents.

#### 3.3.2 Quantitative Linearity

For psychoactive therapeutic drugs, the therapeutic window refers to the concentration range in the blood from the minimum effective concentration (MEC) to the minimum toxic concentration (MTC).^[^ ^43^ ^]^ Within this range, the drugs can exert optimal therapeutic effects while minimizing side effects or toxicity. Therefore, there is a high demand for both the range and accuracy of drug concentration monitoring in clinical settings. According to the consensus guidelines for therapeutic drug monitoring,^[^ ^44^ ^]^ the effective blood concentration ranges for venlafaxine and desvenlafaxine are 50–400 and 100–400 ng mL^−1^, respectively; and the effective blood concentration ranges for risperidone and 9‐hydroxyrisperidone are 10–120 and 15–120 ng mL^−1^, respectively.

The pretreatment device designed in this study has the capacity to process eight samples concurrently, thereby enabling the establishment of a standard curve using spiked serum at eight concentration points. With fully automated pretreatment, sample introduction, MS analysis, and intelligent data processing, the system can automatically complete standard curve generation within 30 min in a single run, improving efficiency by at least 2–3 times over traditional LC‐MS/MS methods. The linear quantification curves for the four drugs are shown in Figure 5C–F. Based on the detection performance of the instrument and taking into account individual variability and different clinical scenarios, the linear quantification ranges developed in this study were set at 10–1280 ng mL^−1^ for venlafaxine and desvenlafaxine, and 2.5–320 ng mL^−1^ for risperidone and 9‐hydroxyrisperidone, which cover the effective therapeutic concentration ranges of these drugs. The corresponding correlation coefficients (R^2^) for the four drugs are 0.9972, 0.9969, 0.9966, and 0.9981, indicating that our standard curves exhibit good linearity and that the system demonstrates excellent response performance across different concentration ranges.

#### 3.3.3 Accuracy and Precision

In clinical testing, accuracy is a critical parameter that reflects the closeness of a measured value to the true value, directly impacting the credibility of the test results. Precision, on the other hand, indicates the consistency of a system in repeated measurements and serves as a fundamental indicator of the method's stability and reliability.

To comprehensively evaluate the accuracy and precision of our system, we conducted back‐calculation tests on four spiked psychoactive drugs. Each drug was tested at two concentration levels (venlafaxine and desvenlafaxine: 960 and 60 ng mL^−1^; risperidone and 9‐hydroxyrisperidone: 240 and 15 ng mL^−1^). The experiments were carried out over five consecutive days, with one batch analyzed in the morning and another in the afternoon each day. In each batch, two replicates were measured at each concentration level, forming a 5 × 2 × 2 study design. All data were recorded sequentially and analyzed using standard statistical methods. Detailed results are provided in the supplementary Tables S3,S4 (Supporting Information).

For accuracy assessment, back‐calculated concentrations were compared with the theoretical spiked values to determine the deviation at each concentration level. The results showed that venlafaxine and desvenlafaxine had back‐calculated biases of 0.74% and 7.98% at high concentrations, and 2.19% and 4.72% at low concentrations, respectively. Risperidone and 9‐hydroxyrisperidone exhibited biases of 2.28% and 8.20% at high concentrations, and 0.30% and 1.12% at low concentrations, respectively. The overall mean accuracy for the four drugs was 3.46%, all within the clinically acceptable threshold of ±10%, indicating good quantitative accuracy of the method.

For precision assessment, the relative standard deviation (RSD) of measurements across different batches over five days was evaluated. The RSDs for venlafaxine and desvenlafaxine were 7.16% and 7.67% at high concentrations, and 7.10% and 9.33% at low concentrations, respectively. For risperidone and 9‐hydroxyrisperidone, the RSDs were 5.34% and 6.04% at high concentrations, and 4.73% and 5.27% at low concentrations, respectively. All values met the clinical requirement of an RSD below 15%, further confirming the good repeatability and robustness of the method.

### 3.4 Clinical Comparative Test

**Figure 6:**
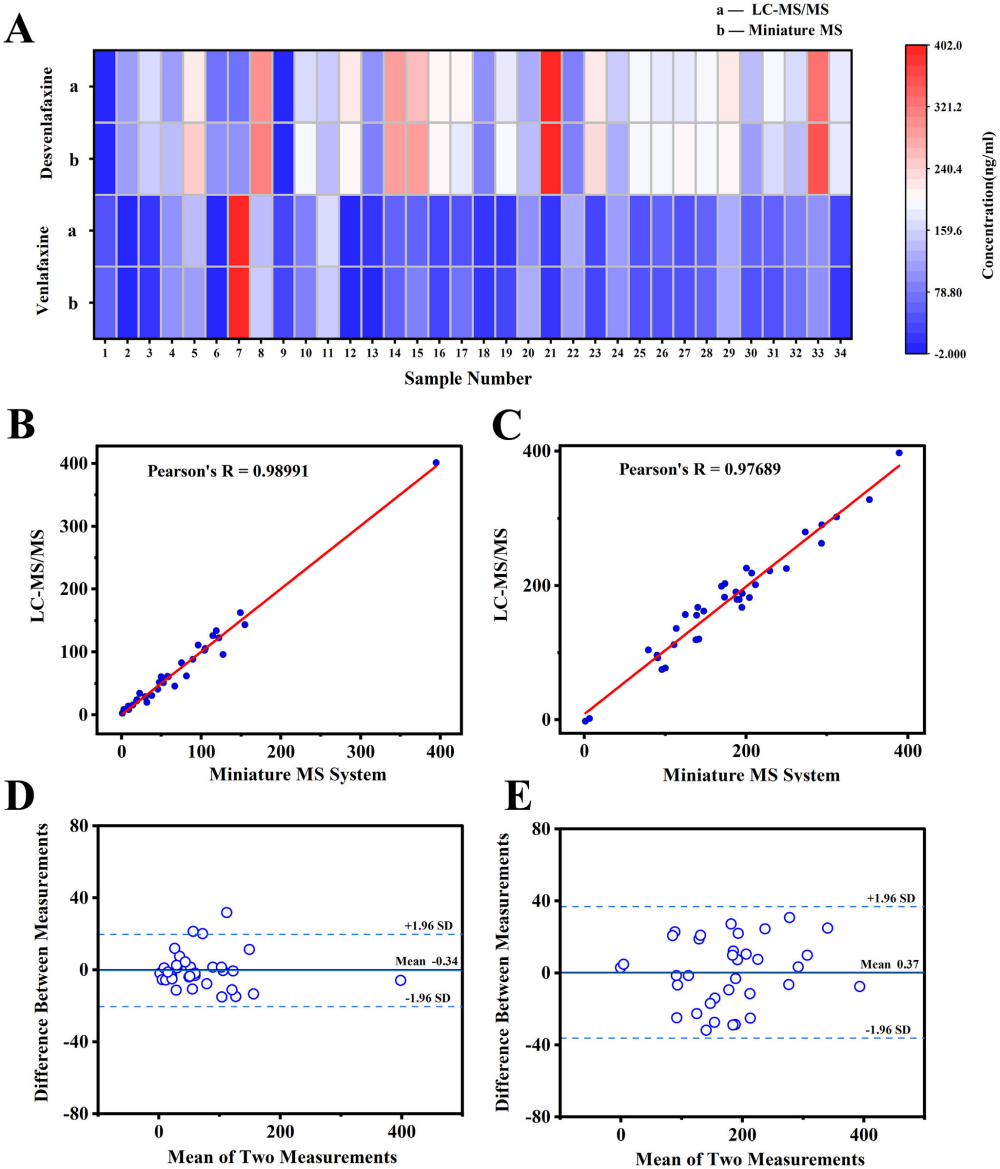
Clinical comparison results of the system in this study against LC‐MS/MS. A) Heatmap of test results for 34 clinical samples from patients taking venlafaxine; B,C) Bland‐Altman consistency analysis for venlafaxine and desvenlafaxine; D,E) Pearson correlation analysis for venlafaxine and desvenlafaxine.

Preliminary experiments have successfully identified the critical indicators for the effective monitoring of blood drug concentrations using the recently developed system. To further refine the practical clinical applications of this technology, a comparative validation experiment was conducted using clinical samples from 35 patients treated with venlafaxine. Serum samples from each patient were separated into two portions. Internal standards of venlafaxine‐d6 and desvenlafaxine‐d6 were then added to these samples. Following this step, both the integrated miniature system and the traditional LC‐MS/MS method were employed for quantitative analysis. And the test results are shown in **Figure** **6**A.

Pearson correlation analysis and Bland‐Altman consistency analysis were performed on the results from the two methods. As demonstrated in Figure 6B,C, the Pearson correlation coefficients for measuring venlafaxine and desvenlafaxine were 0.99 and 0.98, respectively, indicating a high degree of agreement between the two methods when measuring the same batch of samples. And Bland‐Altman analysis revealed systematic mean differences of 0.34 ng mL^−1^ (venlafaxine) and 0.36 ng mL^−1^ (desvenlafaxine) between the two measurement methods. The limits of agreement for venlafaxine and desvenlafaxine were established as −35.61 to 36.35 and −20.13 to 19.45 ng mL^−1^, respectively. As illustrated in Figure 6D,E, the disparity in measurement outcomes between the two methodologies falls within the 95% limits of agreement.

These results underscore the reliability and precision of the integrated miniaturized blood processing and mass spectrometry analysis system, which demonstrates strong potential for practical clinical application. The system's high throughput enables rapid calibration and concentration determination for eight samples within 30 min, streamlining analytical workflows. Its fully automated design reduces operational complexity, making it user‐friendly for medical staff with minimal training requirements. In contrast to costly LC‐MS/MS instruments, this system offers superior cost‐effectiveness, effectively meeting the testing demands of primary healthcare settings. Of course, limited by the magnetic nanoparticles, the method presented in this study is currently only effective for detecting low‐polarity drug molecules. And future refinements in magnetic solid‐phase extraction are expected to expand its analytical scope.

## 4 Conclusion 

Mass spectrometry has gradually become a standard clinical method for the detection of small molecule biomarkers in biological fluids. This study develops an integrated miniaturized blood processing and analysis system (imBPMS) to address key challenges in clinical MS, including automation, intelligence, miniaturization, and high throughput. The system combines magnetic solid‐phase extraction with a miniature mass spectrometer and integrates a U‐net deep learning algorithm for intelligent peak identification, enabling a fully automated workflow, from sample pretreatment to MS analysis and intelligent quantitative data processing.

By optimizing parameters such as the amount of magnetic nanoparticles and the washing/elution protocols, the system achieves comparable process efficiency to traditional manual liquid–liquid extraction using fewer reagents. Each run can establish quantitative curves and process eight samples in parallel within 30 min, significantly improving detection efficiency. For analysis, a dual‐target ion parallel tandem mass spectrometry analysis method was employed to eliminate errors from separate injections of target and internal standard ions. Additionally, the U‐net algorithm was introduced to accurately identify peak areas, enabling precise quantification of small‐molecule drugs. The quantitative curves for venlafaxine, desvenlafaxine, risperidone, and 9‐hydroxyrisperidone all achieved correlation coefficients (R^2^) above 0.99, with quantification ranges covering clinically required levels. The average accuracy based on back‐calculated concentrations was 3.46% and precision tests yielding RSDs below 10%. Furthermore, the results also showed good consistency with existing clinical LC‐MS methods.

Compared to traditional approaches, imBPMS offers fully automated analysis from sample loading to result output, minimizing human error while maintaining portability and high precision. It demonstrates strong potential to advance precision medicine, particularly in individualized therapeutic monitoring and point‐of‐care diagnostics. In the future, this platform could be extended to qualitative and quantitative detection of small‐molecule biomarkers in various biological fluids, further advancing the clinical application of mass spectrometry in precision medicine.

## Conflict of Interest

The authors declare no conflict of interest.

## Author Contributions

Y.Z. and J.A. contributed equally to this work and share the first authorship. Y.Z., J.Z., and X.H. designed the research and wrote the manuscript; Y.Z., M.L., and J.L. performed the investigation; Z.Y. and K.C. set up the device; J.A. developed the algorithm; Z.Z. and S.X. conducted the clinical comparative experiments; B.Z., S.X., J.Z., and X.H. provided research guidance and project support.

## Supporting information



Supporting Information

## Data Availability

The data that support the findings of this study are available from the corresponding author upon reasonable request.
