# Supplementary material for: Fully Automated Deep Learning Enabled Miniature Mass Spectrometry System for Psychoactive Therapeutic Drug Monitoring
Source: Adv Sci (Weinh). 2025 May 23;12(32):e02721. doi: 10.1002/advs.202502721 (PMC12407256; doi:10.1002/advs.202502721)
Supplement: Supplementary file 1 — Supporting Information [file ADVS-12-e02721-s001.docx]

**Supplementary Information**

**Fully Automated Deep Learning Enabled Miniature Mass Spectrometry System for Psychoactive Therapeutic Drug Monitoring**

Yuanhao Zhou^1,2^, Zi Ye^1,2^, Jiawen Ai^3,4^, Kexin Chen^1,2^, JingXiong Lin^1,2^, Zhenhua Zhang^5,6^, Mi Luo^7^, Benjie Zhou^6^, Shijian Xiang^6*^, Jianhua Zhou^1,2*^, Xinming Huo^1,2*^

1. School of Biomedical Engineering, Shenzhen Campus of Sun Yat-sen University, Shenzhen 518107, China
2. Key Laboratory of Sensing Technology and Biomedical Instruments of Guangdong Province, School of Biomedical Engineering, Sun Yat-sen University, Guangzhou 510275, China
3. Division of Advanced Manufacturing, Graduate School at Shenzhen, Tsinghua University, Shenzhen 518055, China
4. State Key Laboratory of Precision Measurement Technology and Instruments, Department of Precision Instrument, Tsinghua University, Beijing 100084, China
5. School of Pharmaceutical Sciences (Shenzhen), Shenzhen Campus of Sun Yat-sen University, Shenzhen, 518107, China.
6. Shenzhen Key Laboratory of Chinese Medicine Active Substance Screening and Translational Research, Department of Pharmacy, The Seventh Affiliated Hospital, Sun Yat-sen University, Shenzhen, 518107, China
7. CHIN Instrument (Hefei) Co., Ltd., Hefei 231200, China

* Corresponding Author: xiangshj3@mail.sysu.edu.cn, zhoujh33@mail.sysu.edu.cn, [huoxm@mail.sysu.edu.cn](mailto:huoxm@mail.sysu.edu.cn,)

Figure S1 Schematic diagram of the microstructure of magnetic nanoparticles.


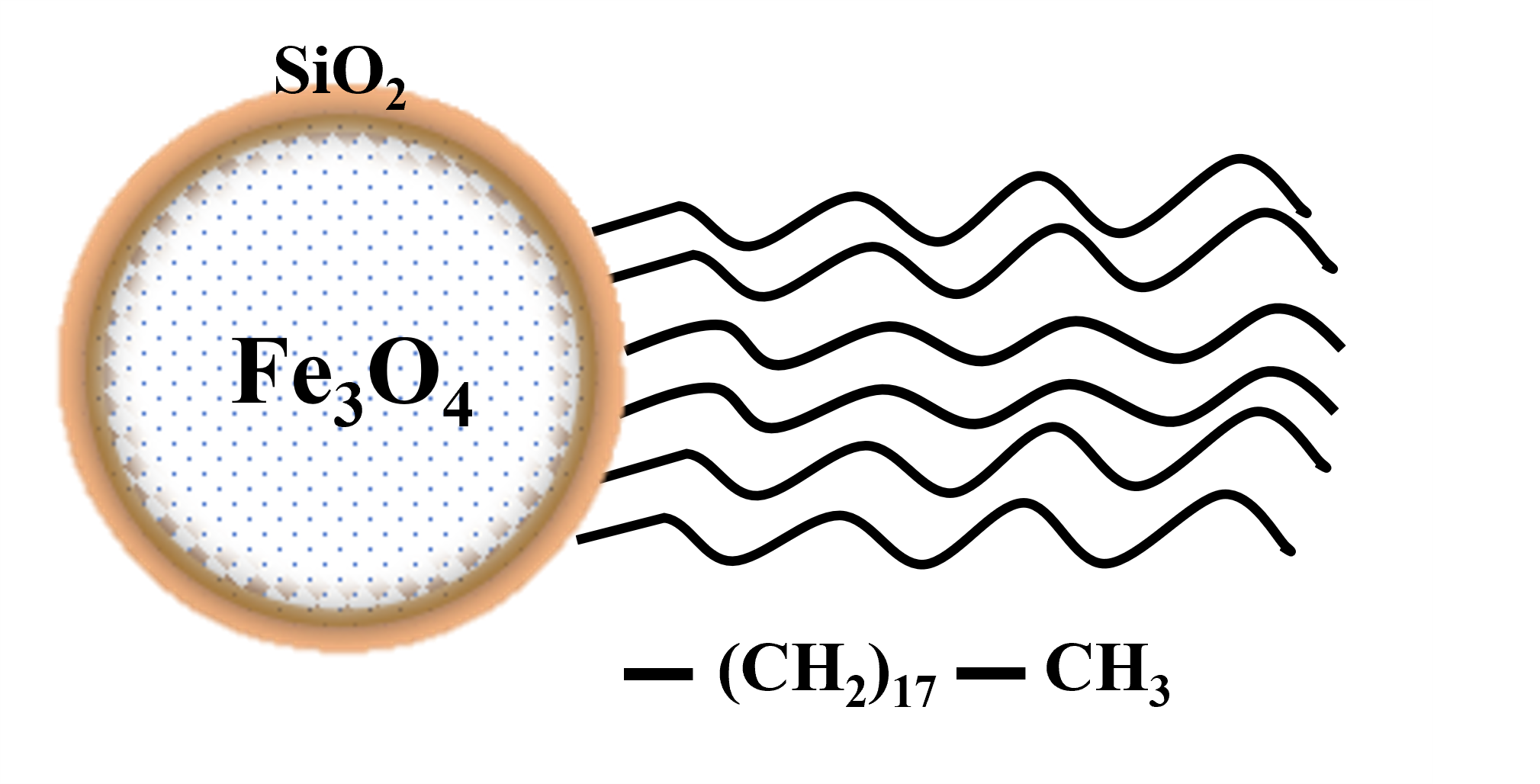


Figure S2 Baseline drift phenomenon when using pure methanol as eluent solvent.


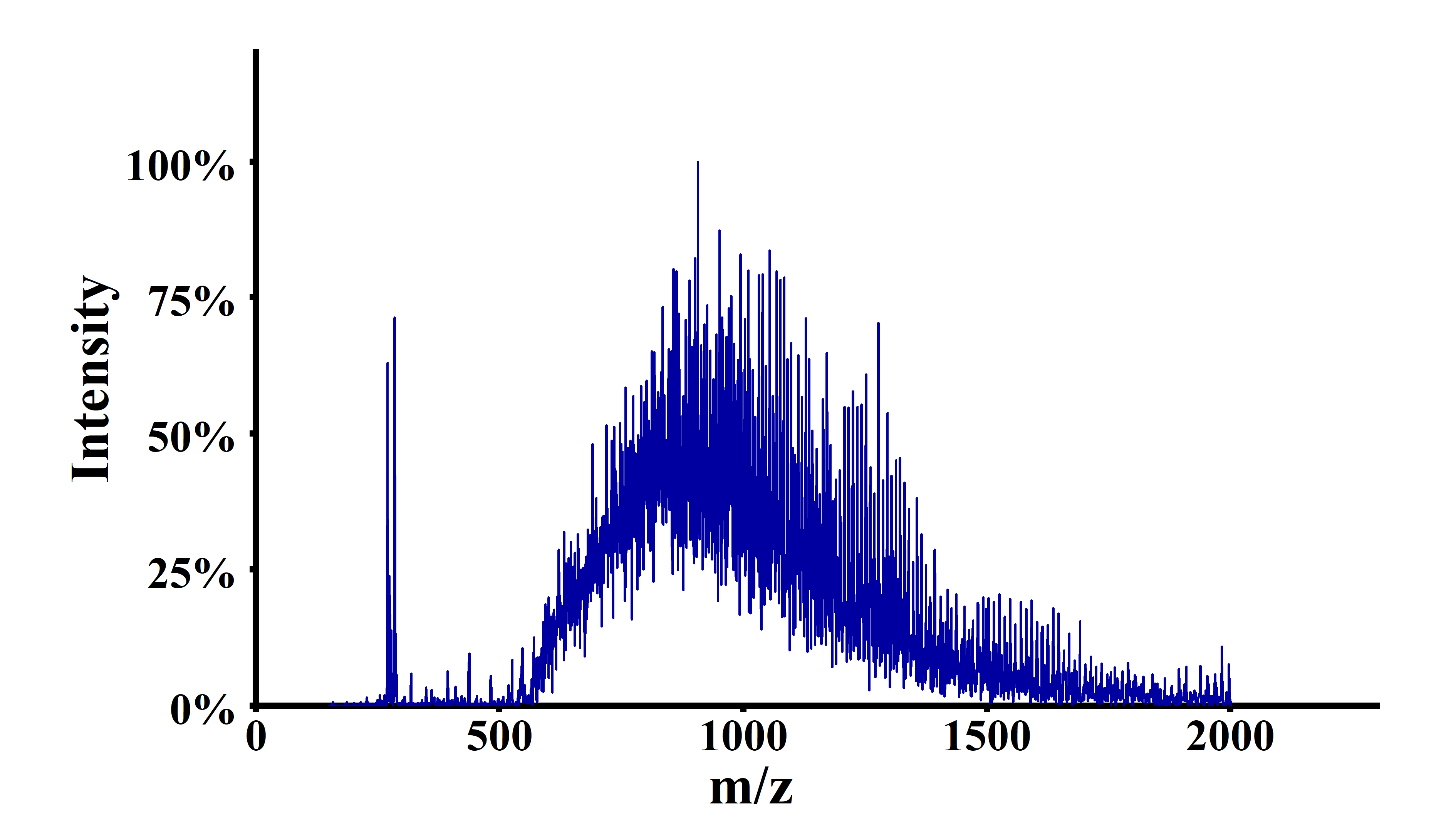


Figure S3 Three common types of mass spectrum peaks of the characteristic fragment ion for venlafaxine collected by the miniature mass spectrometer. A: Normal peak shape spectrum, which has a normal distribution shape; B: Tailing peak shape spectrum, which is affected by unstable resonance excitation or high pressures; C: Split peak shape spectrum, which is affected by baseline drift or noise interference.


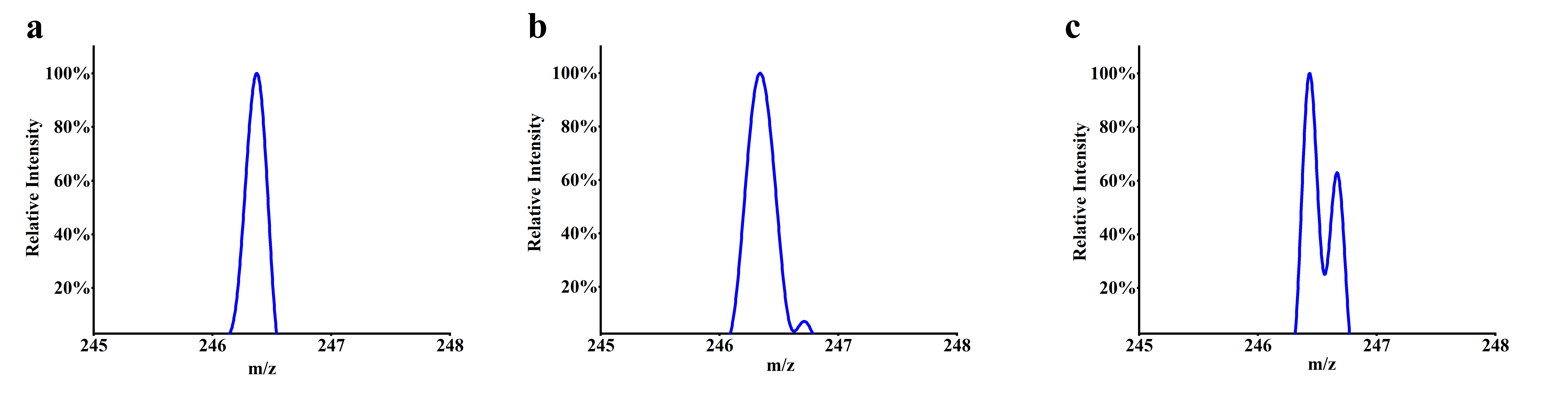


Table S1 Chemical and MS information of the selected psychoactive therapeutic drugs.

| **Drug name** | **CAS Registry Number** | **Molecular formula** | **Relative molecular mass** | **Precursor ion (*m/z*)** | **Characteristic Fragment Ion (*m/z*)** |
| --- | --- | --- | --- | --- | --- |
| Venlafaxine | 93413-69-5 | C_17_H_27_NO_2_ | 277.38 | 278 | 246 |
| Desvenlafaxine | 93413-62-8 | C_16_H_25_NO_2_ | 263.38 | 264 | 260 |
| Risperidone | 106266-06-2 | C_23_H_27_FN_4_O_2_ | 410.48 | 411 | 191 |
| 9-hydroxy risperidone | 144598-75-4 | C_23_H_27_FN_4_O_3_ | 426.48 | 427 | 207 |
| Venlafaxine-d6 | 1020720-02-8 | C₁₇H₂₁D₆NO₂ | 283.38 | 284 | 252 |
| Desvenlafaxine- d6 | 1062605-69-9 | C₁₆H_19_D₆NO₂ | 269.38 | 270 | 266 |
| Risperidone- d4 | 1020719-76-9 | C_23_H_23_D_4_FN_4_O_2_ | 414.48 | 415 | 195 |
| 9-hydroxy risperidone- d4 | 1020719-55-4 | C_23_H_23_D_4_FN_4_O_3_ | 430.48 | 431 | 211 |

Table S2 Calculation method for evaluating the performance of the screening strategy.

| **Sample situation** | **Test results** | | **Sum** |
| --- | --- | --- | --- |
|  | **Positive** | **Negative** |  |
| Positive | $N_{11}$ | $N_{12}$ | $N_{1.}\text{=}N_{11}+N_{12}$ |
| Negative | $N_{21}$ | $N_{22}$ | $N_{2.}\text{=}N_{21}+N_{22}$ |
| Sum | $N_{.1}\text{=}N_{11}+N_{21}$ | $N_{.2}\text{=}N_{12}+N_{22}$ | $N=N_{1.}+N_{2.}=N_{.1}+N_{.2}$ |
| Significant difference ($x^{2}$) | $x^{2}=(\vert N_{12}-N_{21}\vert-1)^{2}/(N_{12}+N_{21})$  Degree of freedom $\left(df\right)=1$ | | |
| Sensitivity($p_{+}$, %） | $p_{+}=N_{11}/N_{1.}$ | | |
| Specificity（$p_{-}$, %） | $p_{-}=N_{22}/N_{2.}$ | | |
| False negative rate ($pf_{-}$, %) | $pf_{-}=N_{12}/N_{1.}=100-p_{+}$ | | |
| False positive rate ($pf_{+}$, %) | $pf_{+}=N_{21}/N_{2.}=100-p_{-}$ | | |
| Relative accuracy，% | $(N_{11}+N_{22})/(N_{1.}+N_{2.})$ | | |

**Table S3** Back-calculated accuracy of drug concentration measurements over five days (morning and afternoon sessions) at low concentration levels for the four psychoactive drugs.

| **day** | **Time of Day** | **Risperidone**  **(ng/ml)** | **9-hydroxy risperidone**  **(ng/ml)** | **Venlafaxine**  **(ng/ml)** | **Desvenlafaxine**  **(ng/ml)** |
| --- | --- | --- | --- | --- | --- |
| 1 | AM | 14.70 | 15.14 | 58.90 | 56.49 |
|  | AM | 14.61 | 15.87 | 63.52 | 61.74 |
|  | PM | 14.66 | 14.87 | 61.25 | 59.13 |
|  | PM | 15.06 | 14.90 | 65.85 | 64.43 |
| 2 | AM | 14.10 | 15.68 | 60.58 | 60.32 |
|  | AM | 14.10 | 15.23 | 56.89 | 60.95 |
|  | PM | 16.10 | 14.55 | 64.79 | 65.55 |
|  | PM | 15.83 | 15.64 | 58.78 | 66.82 |
| 3 | AM | 14.74 | 16.77 | 69.01 | 61.76 |
|  | AM | 14.84 | 15.01 | 56.66 | 67.81 |
|  | PM | 16.07 | 14.02 | 57.71 | 54.64 |
|  | PM | 14.99 | 15.80 | 67.82 | 55.25 |
| 4 | AM | 15.39 | 13.91 | 59.26 | 69.65 |
|  | AM | 14.98 | 15.57 | 63.14 | 62.15 |
|  | PM | 17.09 | 15.03 | 61.56 | 64.39 |
|  | PM | 15.83 | 15.87 | 61.90 | 65.37 |
| 5 | AM | 14.08 | 14.35 | 65.98 | 62.95 |
|  | AM | 15.10 | 14.64 | 64.54 | 80.19 |
|  | PM | 14.36 | 14.83 | 56.20 | 61.44 |
|  | PM | 14.35 | 15.98 | 51.97 | 55.55 |
| Mean | | 15.05 | 15.18 | 61.32 | 62.83 |

**Table S4** Back-calculated accuracy of drug concentration measurements over five days (morning and afternoon sessions) at high concentration levels for the four psychoactive drugs.

| **day** | **Time of Day** | **Risperidone**  **(ng/ml)** | **9-hydroxy risperidone**  **(ng/ml)** | **Venlafaxine**  **(ng/ml)** | **Desvenlafaxine**  **(ng/ml)** |
| --- | --- | --- | --- | --- | --- |
| 1 | AM | 243.10 | 263.45 | 933.39 | 1047.24 |
|  | AM | 242.10 | 260.31 | 1101.49 | 1019.95 |
|  | PM | 241.15 | 252.99 | 998.01 | 1050.15 |
|  | PM | 228.26 | 246.54 | 974.52 | 1104.67 |
| 2 | AM | 238.25 | 252.57 | 975.50 | 1113.78 |
|  | AM | 239.62 | 267.22 | 1013.01 | 911.34 |
|  | PM | 230.89 | 285.25 | 1017.05 | 929.12 |
|  | PM | 273.39 | 267.87 | 911.27 | 1113.88 |
| 3 | AM | 229.26 | 276.24 | 965.82 | 945.70 |
|  | AM | 235.19 | 242.60 | 971.93 | 974.71 |
|  | PM | 247.04 | 247.01 | 1081.11 | 1136.16 |
|  | PM | 248.54 | 246.04 | 1007.33 | 974.31 |
| 4 | AM | 270.15 | 251.45 | 995.12 | 1054.55 |
|  | AM | 279.66 | 263.92 | 1048.54 | 1029.76 |
|  | PM | 246.08 | 242.08 | 953.98 | 916.04 |
|  | PM | 239.35 | 281.03 | 856.10 | 1073.25 |
| 5 | AM | 235.21 | 239.94 | 897.52 | 1021.11 |
|  | AM | 258.60 | 279.83 | 873.67 | 1151.31 |
|  | PM | 230.44 | 263.56 | 905.30 | 994.01 |
|  | PM | 253.40 | 263.90 | 861.99 | 1171.65 |
| Mean | | 245.48 | 259.69 | 967.13 | 1036.64 |
